# A Case Report of Implant Fracture of Extensively Porous-Coated, Distally Fixated Cementless Long Stem: Detailed Course of Stem Bending Development

**DOI:** 10.1155/2015/895214

**Published:** 2015-05-21

**Authors:** Tomohiro Mimura

**Affiliations:** Department of Orthopedics Surgery, Shiga University of Medical Science, Shiga 520-2192, Japan

## Abstract

We report the fracture of a cementless long stem in a distally fixated, extensively porous-coated femoral prosthesis used for revisional total hip arthroplasty (THA) 9 years previously in a 48-year-old woman, measuring 58 kg in weight and 155 cm in height. Following resolution of an infection 7 years after the revisional THA, a series of posterior dislocations occurred up until 7 months before sudden stem fracture. Extensive radiographic imaging evidence indicated a gradual progression of bending in the stem, and scanning electron microscope energy dispersive X-ray spectroscopy revealed oxygen in the medial and lateral sections, suspecting in vivo corrosion. We retrospectively examined the detailed course of stem bending development prior to stem fracture. The stem bending immediately after the revisional THA, at the first dislocation, and immediately before the stem fracture was 0°, 1.9°, and 5.2°, respectively. We consider that the cyclic loading with poor proximal bone support, distal fixation, and small diameter were the potential risk of this implant fracture. However, the course of stem bending development suggested that repeated operations and several dislocations might have aggravated the implant fatigue in the present case.

## 1. Introduction

A fracture of the femoral component of an implant after total hip arthroplasty (THA) is not a common complication. Charnley reported a rate of 0.23% for femoral stem fractures in 1975 [1], while the Mayo Clinic reported that the incidence of femoral component fracture after THA was 0.6% [2]. However, the American Association of Hip and Knee Surgeons reported the prevalence of stem fractures to be 0.27% [3]. Several studies have investigated fractures of the femoral component after THA, most of which have been noted to occur with a cemented stem [4–6], with fewer published reports of cementless stem fracture [7–9]. In this study, we report the case of a cementless stem fracture that occurred after revisional THA involving an extensively porous-coated and distally fixated cementless long stem. We also evaluated a series of radiographic images obtained prior to the stem fracture. To the best of our knowledge, this report is the first to highlight continuous bending development prior to stem fracture.

## 2. Case Presentation

**Figure 1:**
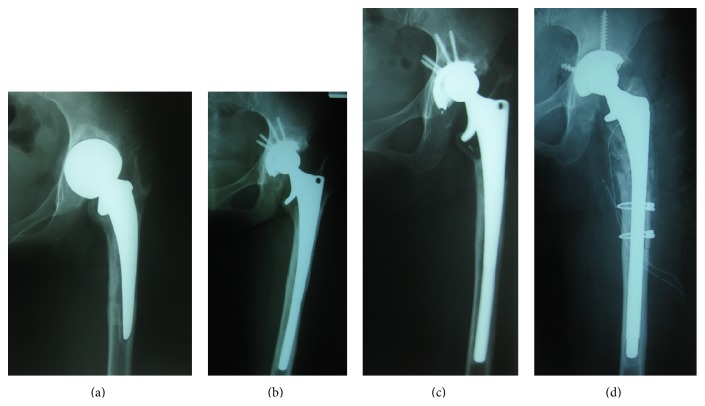
(a) Bipolar hemiarthroplasty (BHR) loosened aseptically and migrated superiorly. (b) Total hip arthroplasty (THA) was performed as a conversion from BHR to THA 22 years ago. Cup was unknown (Zimmer, Warsaw, IN, USA) and stem was BIAS long stem (Zimmer). (c) Stem prosthesis of primary THA loosened aseptically. (d) Revisional THA (cup, trilogy, stem, versis beaded full coat plus; inner head, 26 mm cobalt-chrome) (Zimmer) was performed because of aseptic loosening of THA 9 years ago.

The patient was a 48-year-old woman, measuring 58 kg in weight and 155 cm in height, with a body mass index (BMI) of 24 kg/m^2^. She underwent a revisional THA for aseptic loosening, 9 years after which a stem fracture occurred. The case history was complex. First, she received a compression hip screw (CHS) for an intertrochanteric fracture suffered in a traffic accident at the age of 20, which was soon followed by a bipolar hemiarthroplasty (BHR) procedure because of failure of the CHS. Thereafter, aseptic loosening of the BHR was noted and she underwent conversion from BHR to a cementless THA (Zimmer, Warsaw, IN, USA) at the age of 26 (Figures 1(a) and 1(b)). At 39 years of age, a revisional THA (Trilogy cup, versis beaded full coat plus stem, 26 mm cobalt-chrome inner head, Zimmer) was performed because of aseptic loosening of the previous THA (Figures 1(c) and 1(d)). This cobalt-chrome alloy stem was extensively porous-coated, with a diameter of 12 mm and length of 200 mm. During the operation, a strut allograft bone graft was required because of a comminuted fracture in the proximal portion of the femur that occurred during extraction of the stem, even though an extended trochanteric osteotomy (ETO) was performed. After the operation, the comminuted fragments and strut allograft were gradually absorbed. Additionally, a late acute homogeneous infection occurred 7 years after the revisional THA. The infection completely resolved following two operations for debridement and application of antibiotics-loaded cement beads, and the prosthesis was preserved. Thereafter, posterior dislocations occurred several times, the final one 7 months before the stem fracture.

**Figure 2:**
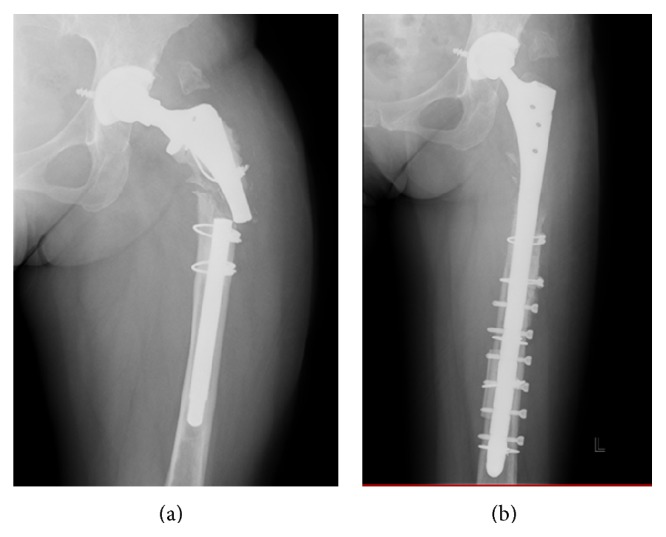
(a) During walking with a single axillary crutch, the stem was suddenly fractured at the site of well-fixated proximal level. (b) Re-revisional THA was performed with Delta-lock system (stem, titanium-6 aluminum-4 vanadium alloy; head, 26-mm Cobalt-chrome-molybdenum alloy (Nakashima Medical Co. Ltd., Okayama, Japan)); cup was not changed.

**Figure 3:**
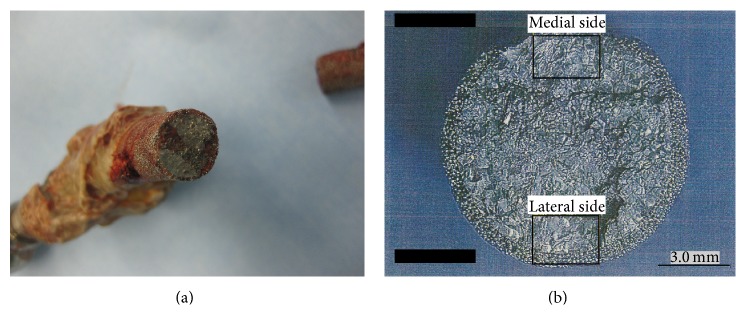
(a) The picture of retrieved implant. Lower left is medial side of the implant. (b) Extended section picture of proximal part. There was a clear step in the medial side, suggesting that lateral side was the starting point of fracture and medical side was lastly fractured.

Two years later after the infection was finally healed, the stem was suddenly fractured while the patient was walking with a single axillary crutch (Figure 2(a)). She underwent a re-revisional THA with a Delta-lock system (Nakashima Medical, Okayama, Japan) without a change of cup (Figure 2(b)). The section pictures of retrieved implant revealed a clear step in the medial side, suggesting that medial side was lastly fractured (Figures 3(a) and 3(b)). The new titanium-6 aluminum-4 vanadium alloy stem diameter was 14 mm and the length was 280 mm, fixed with six screws.

**Figure 4:**
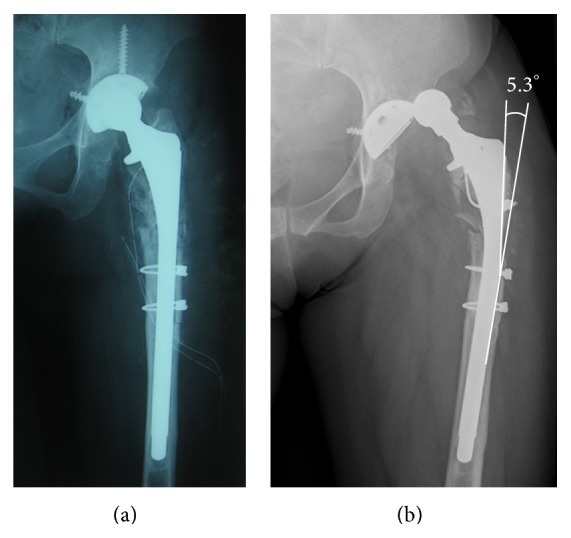
(a) The stem lateral bending was 0° immediately after revisional THA. (b) The stem bending was stronger than before, with the bending degree being 5.3° at final dislocation.

**Figure 5:**
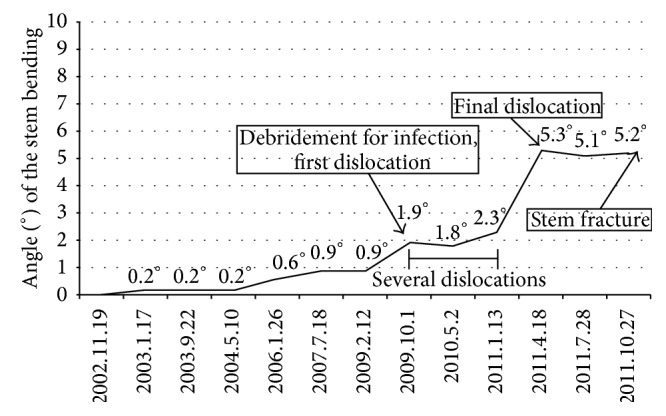
The stem bending was 0° immediately after the revisional THA. The stem bending gradually progressed during first five years. The stem bending immediately after the first dislocation was 1.9° and then the stem bending at final dislocation was 5.3°. The stem bending before the stem fracture was 5.2°.

**Figure 6:**
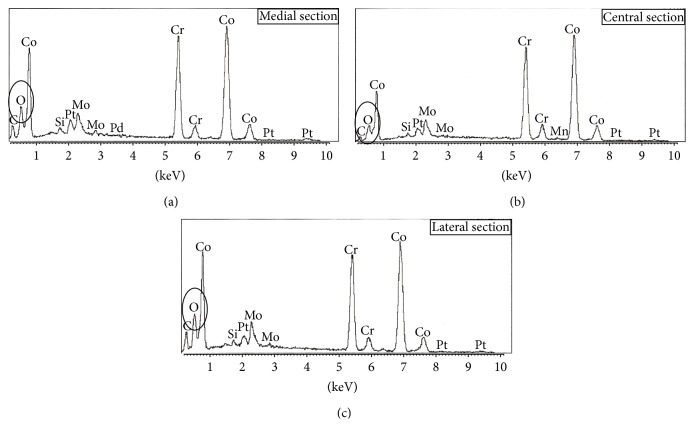
The results of scanning electron microscope- (SEM-) energy dispersive X-ray spectroscopy (SEM-EDX). (a), (b), and (c) showed the results of SEM-EDX at medial section, central section, and lateral section, respectively. Much higher oxygen at the medial and lateral sections was detected compared with the central section.

Immediately after the revisional THA was performed, stem bending was 0° (Figure 4(a)), which gradually progressed (Figure 5). The degree of stem bending immediately after the first dislocation was 1.9°. After the final dislocation, the degree of bending (5.3°) was more pronounced than before (Figures 4(b) and 5). The bending angle immediately before stem fracture was 5.2°. No bending was seen on lateral views obtained by radiography at any time. A scanning electron microscope (SEM) examination showed striation in the medial and lateral sections. SEM-energy dispersive X-ray spectroscopy (SEM-EDX) revealed that there was much higher oxygen at the medial and lateral sections as compared with the central section, suspecting that there had been in vivo corrosion before sudden implant fracture (Figure 6).

## 3. Discussion

We encountered a case of fracture of a cementless long stem in a distally fixated, extensively porous-coated femoral prosthesis. Implant fracture of the femoral component occurred 9 years after the revisional procedure. A stem fracture after THA is not a common complication in Japan either. The manufacturer Zimmer, Japan, has noted four stem fractures (0.3%, 4/1370) in patients with the same type of femoral implant in Japan, including the present case, all of which were revision cases.

Cementless stem fractures are thought to be caused by a weakness of the prosthesis, such as the prosthesis neck [10–12], a narrow stem diameter excluding madreporic corrugation [8], the junction of the madreporic corrugations and smooth plate [13], or narrowness of the anteroposterior dimension and the depth of recess for the titanium mesh pads [14]. Sotereanos et al. reported two cases of cementless stem fracture among 122 patients (1.6%) who received an extensively porous-coated, single-sized cobalt-chrome component, while in their subsequent series no stem fractures were noted (0/227) when stems of multiple size were available, suggesting that inadequate stem thickness increases the risk of fracture [15].

Several authors have reported risk factors related to a cementless stem fracture. Kishida et al. noted that lack of proximal support, a champagne-fluted canal, a fully porous stem made of a cast cobalt-chrome-molybdenum alloy, and a narrow stem core were factors contributing to such a fracture [8]. Busch et al. also reported stem fractures in 2.3% of 219 revisional THA cases that used a cementless extensively porous-coated and distally fixated femoral stem, as in the present case, and considered that risk factors were poor proximal bone support, BMI > 30, small stem diameter (<13.5** **mm), and use of an extended trochanteric osteotomy [7]. They recommended use of a strut allograft in conjunction with an ETO in patients with poor proximal femoral bone stock. Similarly, Carrera et al. reported the 2% stem fracture (2/100) using cement less distal clocking stem for revisional THA [9]. They also reported that risk factors for stem fracture were obese (BMI > 30), poor metaphyseal bone support, poor initial diaphyseal filling, and small diameter (12** **mm). Both patients underwent re-revisional implantations with 14** **mm or 16** **mm stems.

In our case, we considered that the cyclic loading with poor proximal bone support, distal fixation, and small diameter (12 mm) were the potential risk of this implant fracture the same as previous reports, because the development course of stem bending had revealed that the bending have been immediately and gradually occurred after implantation. However, we supposed that the repeated operations and dislocations might have aggravated the implant fatigue, as the stem bending got worse after debridement and subsequent several dislocations. We also supposed that two-times debridement (direct curettage to prosthesis) might accelerate the in vivo corrosion suspected by SEM-EDX examination. Prediction of stem fracture from the bending angle is not easy, because this angle is affected by the stem length of the nonfixation area and the level of rigid fixation. However, when deterioration of stem bending occurs, it may indicate a forthcoming stem fracture attributable to sudden fatigue.

With the new femoral stem now in our patient, careful attention will be given to avoidance of possible refracture of the stem. Her activities of daily living are nearly 2/3 partial weight-bearing with an axillary crutch or Lofstrand crutch. She always uses crutches in situation of indoor as well as outdoor. It will also be important to examine further X-ray images for bending over an extended follow-up period, because the condition of no proximal bone support remains.
